# Thymic central tolerance takes centre stage in autoimmune disease

**DOI:** 10.1016/j.lanwpc.2025.101638

**Published:** 2025-07-12

**Authors:** Smaila Mulic-Al Bunni, Adam E. Handel

**Affiliations:** Nuffield Department of Clinical Neurosciences, University of Oxford, Oxford

The thymus functions as the primary lymphoid organ responsible for the generation of T cells and plays a crucial role in preventing autoimmune reactions by eliminating autoreactive cells before release into the peripheral T cell pool. It is largest and most active during the neonatal and pre-adolescent periods, after which it undergoes a process of involution that continues for the remainder of life.^1^ This thymic involution is characterised by a reduction in tissue mass, cellularity, and structural organisation, resulting in decreased output of naïve T cells, alterations in the peripheral T cell pool,^1^ and ultimately, an age-related decline in immune function.^2^ Thymic pathology can therefore precipitate autoimmunity; indeed, thymomas are well recognised for their association with systemic and neurological autoimmune disorders, most notably myasthenia gravis (MG).^3^

The study conducted by Chang et al. published in *The Lancet Regional Health–Western Pacific* represents the largest cohort to date examining CT-based thymic parameters in the context of 16 autoimmune diseases: systemic lupus erythematosus, Sjögren's syndrome, multiple sclerosis, rheumatoid arthritis, systemic sclerosis, Hashimoto's thyroiditis, ankylosing spondylitis, type 1 diabetes mellitus, myelin oligodendrocyte glycoprotein antibody-associated disease, clinically isolated syndromes, autoimmune hepatitis, autoimmune encephalitis, psoriasis, anti-neutrophil cytoplasmic antibody-associated vasculitis, MG and neuromyelitis optica spectrum disorder.^4^ The authors show that multiple measures of thymic size and morphology differ significantly between patients with various autoimmune diseases and matched healthy controls. Strikingly, thymic involution is consistently more pronounced across the wide range of autoimmune conditions studied.

T-cell-receptor excision circles (TREC) are produced during T cell development in the thymus as part of T cell receptor recombination. Peripheral T cell TREC frequencies can be used as an indicator of thymic output. In healthy individuals, thymic size correlates significantly with TREC frequencies, suggesting that assessment of thymic size acts as a proxy for some measures of thymic output.^5^ Thymic size, however, is not always indicative of its functional output. In HIV-infected individuals, for example, no clear correlation exists between CT-defined thymic volume and thymic output. In these individuals, reduced absolute numbers of naïve T cells were observed regardless of thymic size, age, TREC levels, or antiretroviral therapy.^6^ These findings suggest that thymic size is only a semi-quantitative indicator and cannot reliably reflect true thymic output.^7^ Indeed, given that the thymus produces both effector and regulatory T cells, the effects of altered thymic output of peripheral T cell autoreactivity will depend on the precise nature of the emigrant T cells. Further research is needed to more precisely correlate macroscopic and microscopic thymic structure with functional output.

Beyond chronological ageing, a variety of factors, including pro-inflammatory cytokines and hormonal changes, can trigger acute thymic atrophy, a transient state characterised by a temporary loss of thymic cellularity and structure, yet with the potential for full recovery once the trigger is removed.^8^ In the context of autoimmune disease, immunotherapeutic interventions, particularly glucocorticoid treatment, can lead to acute thymic atrophy. Dexamethasone, a synthetic glucocorticoid widely used in clinical practice, induces extensive apoptosis of thymocytes, resulting in a marked reduction in thymic size and cellularity, with significant depletion of developing T cells and thymic epithelial cells.^8^ It would therefore be informative to know whether patients with autoimmune diseases exhibiting pronounced thymic involution had prior or ongoing glucocorticoid exposure; future work will be required to disambiguate the direct effects of autoimmune disease from those induced by immunotherapy. However, the fact that the authors observed consistent trends in type 1 diabetes mellitus, which is generally not treated with immune treatments, would argue for a disease-specific impact on thymic size.

This study is particularly important in focusing on the potential translational impact of thymic immunobiology in autoimmunity. Outside of the context of some specific diseases, such as myasthenia gravis, the thymus is relatively understudied. It is likely that early negative trials of thymectomy in some autoimmune disease, such as multiple sclerosis,^9^ along with the established nature of thymic involution seen in adult humans promoted a degree of clinical nihilism. However, the combination of an increasingly high resolution understanding of thymic immunobiology, including the recognition of “mimetic” thymic epithelial cells as highly specialised antigen-presenting cells along with an appreciation of novel autoantigen presentation pathways in the thymus,^10^^,^^11^ means that this study is ideally timed to trigger a renaissance of translational thymus research in autoimmunity.

In summary, this study highlights the role of the thymus as a broad range of autoimmune diseases and hints at shared pathogenic pathways reflected in accentuated involution. Clarifying how thymic size and architecture relates to functional output in disease cohorts and how variables such as immunotherapy in autoimmune diseases modify this relationship remains a key priority for future research. This study has the potential to catalyse renewed interest in thymic immunobiology as a target for future translational research to prevent or modulate autoimmunity.

## Contributors

SMB and AEH contributed to conception of this commentary. SMB wrote the first version. SMB and AEH revised the manuscript.

## Declaration of interests

AEH is funded by the MRC (MR/X022013/1 and MR/V007173/1), MyAware and the National Institute for Health Research (NIHR) Oxford Health Biomedical Research Centre. AEH has received funding from UCB-Pharma and served on an advisory board for Argenx. SMB is funded by an EAN Research Training Fellowship. The views expressed are those of the authors and not necessarily those of the NHS, the NIHR or the Department of Health. The funders had no input into the preparation of this manuscript.
